# 

**Published:** 1845-07

**Authors:** 


CONTENTS:

Ferruginous pill of Mercury, - 49
Trial for Malpractice, - - 51

BIBLIOGRAPHICAL NOTICES.

On the Anatomy and Diseases of the Urinary and Sexual Organs, containing the Anatomy of the Bladder and Urethra, and the Treatment of the Obstructions to which these passages are liable. By G. J. GUTHRIE, F.R.S. - 53
Summary of the Transactions of the College of Physicians of Philadelphia, from November, 1844, to March, 1845, - - 55
The Buffalo Medical Journal, - 59

PRACTICAL MEDICINE, &C.

On the Use of Large Doses of Quinine.—Report of a Commitee of the Medical Department of the National Institute, on Dr. Buck's Paper "On the Use and Abuse of Medicine," - - 59
Prognosis of Chancre with reference to the probabilities of Secondary Symptoms.—By M. RICORD of Paris, - - 63
The Engrafting of Nerves, - 63
Reduction of Dislocation of Large Joints, by Power derived from Twisted Rope, - - 64
To Readers, Correspondents, &c. 64

